# New York College of Veterinary Surgeons

**Published:** 1895-04

**Authors:** 


### NEW YORK COLLEGE OF VETERINARY SURGEONS.

The New York College of Veterinary Surgeons held its thirty-eighth annual commencement at the Music Hall of Madison Square Garden on Monday evening, March 18, 1895. The hall was well filled. The prayer and benediction were given by the Rev. Eli Quick, Ph.D. The music was given by the Seventh Regiment band. Professor James Law, of Cornell, gave the address (*vide* page 201 of this journal).

The following gentlemen received their diplomas: William Anderson, New York; Ernest Buckley, California; Frederick Bovers, New York; George D. Burton, New York; Leopold Danielson, Norway; Clarence O. Durfee, New York; Edward B. Doyle, New York; Albert D. Edwards, New York; William J. Fredericks, New Jersey; Vernon B. Height, New Jersey; August D. Krahmer, New York; John C. Kingsland, Pennsylvania; George A. Knapp, New York; George P. Kern, New York; M. O'Malley Knott, New York; Thomas H. Lynch, Connecticut; Russel Lawton, Canada; Nelson Moffatt, New Jersey; Edward A. Manuel, Wisconsin; Patrick J. Mahoney, Massachusetts; David C. McGowan, New York; Patrick McInerney, New York; Alfred T. Norman, New York; William H. C. Neff, Pennsylvania; Albert C. Roberts, New York; Rudolph J. Schreiber, New York; John M. Simpson, New York; Frederick L. Stevens, Maine; Joseph Thackeberry, New York; William J. Taylor, New York; Willy E. R. Weyman, New York.

The class officers were: Thomas H. Lynch, *President;* George D. Burton, *Vice-President;* Rudolph J. Schreiber, *Secretary;* George F. Kern, *Treasurer.*

Executive Committee: Frederick Bovers (*Chairman*), Ernest Buckley, John Kingsland, Edward B. Doyle, William J. Fredericks, Patrick J. Mahoney, August D. Krahmer (*Secretary*).

The ushers were: J. Homer Luff, William Price, Jr., James McNeil, William C. Kelly, J. B. Rue, Charles R. Scattergood, John Tjaden, Thomas E. Smith.

The following prizes were awarded: Faculty Prize—for best general examination, a gold medal—to Willy E. B. Weyman. For best general junior examination, a silver medal—to George H. Harrison. Medal for the best examination on Materia Medica—to Edward A. Manuel. Practical examination, $25, before a committee composed of R. S. Huidekoper, M.D., Vet., Thomas Giffen, M.R.C.V.S., and James Turner, D.V.S.—to M. O'M. Knott. Messrs. Krahmer, Manuel, Mahoney, and Norman were also highly commended.

After the commencement a banquet was held at Martini's, when the following toasts were given, and gratifying addresses were made by two of the trustees, Mr. Savage and Judge Guitteau:

Programme—Frederick Bovers, *Toast Master.* "The New York College of Veterinary Surgeons," Hermann M. Biggs, M.D.; "Our Alma Mater," Harry D. Gill, V.S.; "The Professional Man," George P. Biggs, M.D.; "Class of '95," Thomas H. Lynch, V.S.; "Class of '96," Charles R. Scattergood; "Veterinary Practice," Rush Shippen Huidekoper, M.D., Vet.; "The Banquet," John H. Huddleston, M.D.; "The Press," James H. Ferster, V.S.; "New Remedies in Veterinary Practice," August C. Hassloch, V.S.; "The Future," George F. Kern, V.S.

